# The fundamentals of eye tracking part 2: From research question to operationalization

**DOI:** 10.3758/s13428-024-02590-2

**Published:** 2025-01-24

**Authors:** Ignace T. C. Hooge, Antje Nuthmann, Marcus Nyström, Diederick C. Niehorster, Gijs A. Holleman, Richard Andersson, Roy S. Hessels

**Affiliations:** 1 Experimental Psychology, Helmholtz Institute, Utrecht, The Netherlands; 2 Martinus J. Langeveldgebouw, Heidelberglaan 1, 3584 CS Utrecht, The Netherlands; 3 Institute of Psychology, Kiel University, Kiel, Germany; 4 Lund University Humanities Lab, Lund University, Lund, Sweden; 5 Lund University Humanities Lab and Department of Psychology, Lund University, Lund, Sweden; 6 Department of Cognitive Neuropsychology, Tilburg University, Tilburg, the Netherlands; 7 Tobii AB, Danderyd, Sweden

**Keywords:** Eye tracking, Experimental design, Operationalization

## Abstract

In this article, we discuss operationalizations and examples of experimental design in eye-tracking research. First, we distinguish direct operationalization for entities like saccades, which are closely aligned with their original concepts, and indirect operationalization for concepts not directly measurable, such as attention or mind-wandering. The latter relies on selecting a measurable proxy. Second, we highlight the variability in algorithmic operationalizations and emphasize that changing parameters can affect outcome measures. Transparency in reporting these parameters and algorithms is crucial for comparisons across studies. Third, we provide references to studies for common operationalizations in eye-tracking research and discuss key operationalizations in reading research. Fourth, the IO-model is introduced as a tool to help researchers operationalize difficult concepts. Finally, we present three example experiments with useful methods for eye-tracking research, encouraging readers to consider these examples for inspiration in their own experiments.

## Introduction

This article is the second in a series on the fundamentals of eye tracking (see also Hessels et al. (2025); Nyström et al. (2025); Niehorster et al. (2025)). The articles are aimed at individuals who are (one of) the first in their group, company, or research field to use eye tracking, with a focus on all the decisions one may make in the context of an eye-tracking study. Such individuals may come from academia (e.g., psychology, biology, medicine, educational science, computer science), commercial institutions (e.g., marketing research, usability, decision-making) and non-commercial institutions (e.g., hospitals, air traffic control, military organizations). Note that this is not an exhaustive description of the target audience. More experienced eye-tracking researchers may find useful insights in the article series, or may find the article series a useful reference or hub to relevant research. One may either choose to read this article as the second part of the series, but it can also be read on its own.

The study of eye movements spans over more than a century (for historical overviews, see Płużyczka, 2018; Wade, 2007, 2010, 2015; Wade and Tatler, 2005, 2008). Research in this domain encompasses studies that focus directly on the characteristics of eye movements themselves, as well as investigations into the control mechanisms governing eye movements. Furthermore, the study of eye movements finds application in diverse domains, including diagnostics (psychological, psychiatric, and medical), visual perception, attention, cognition, decision-making, reading, art, visual search, scene viewing, marketing research, cartography, usability, expertise, and many more.

This article is concerned with conducting research utilizing eye-tracking technology, focusing on the operationalization of research questions (e.g., the concretization, definition, and quantification of phenomena, variables and or constructs). While this article is designed to be comprehensible on its own, it also serves as a follow-up to Hessels et al. (2025). The latter article is about the link between theory and research questions that can be answered by the use of an eye tracker. Rather than prescribing methodologies for conducting eye-tracking research, we focus on how eye-tracking research can be effectively executed. We adopt a non-dogmatic stance, particularly concerning experimental design, advocating feasibility and effectivity. This article contains information about the operationalizations of many common concepts in eye-tracking research, the operationalization of questions concerning reading in an applied setting and three prototypes for experiments in experimental psychology, cognitive science and the more applied fields. Although the covered examples may not match the reader’s research area of interest, we believe the operationalizations in the examples can inspire other studies wrestling with similar operationalization challenges.

Smart and useful strategies are employed in the extensive field of eye-tracking research to solve the many existing scientific problems (e.g., how is attention employed during driving? what is the best font for children with ADHD?). Some of these strategies are borrowed from the research domains where eye tracking is applied, with many details scattered throughout the eye-tracking literature’s periphery. Our objective is to share several useful operationalizations of research questions with researchers who may not possess an understanding of the exhaustive eye-tracking literature. Many of the operationalizations discussed originate from the general eye-tracking literature but also from the authors’ own research, scientific collaborations and inquiries into experimental challenges encountered during their roles as instructors in various eye-tracking courses.

In this article, we limit ourselves mostly to video-based eye-tracking technology, as it is currently by far the most commonly used technique. This technique is popular due to the relative affordability and ease of application. The latter makes it also popular in new research fields without a lot of technical and empirical background where investigators are utilizing an eye tracker for the first time.

The primary audience for this article comprises researchers new to the domain of eye tracking, particularly those who may be the sole individuals pioneering such research within their respective groups. This includes early-career researchers such as Ph.D. candidates and master students, as well as more experienced researchers incorporating eye tracking into their repertoire of research methods. Additionally, we posit that this article is also relevant for more experienced researchers who maintain a primary interest in their specific research domains (e.g., cartography, developmental psychology, language studies, archeology, or clinical psychology), for whom eye tracking only serves as a research tool for a select number of studies. We aspire for this article to serve as a shortcut or hub to effective and efficient eye-tracking research methods for such researchers.

We believe that acquiring complex and new knowledge is often an iterative process. A novice reader might go through this article from start to finish, and consult the cited literature for any new or unclear concepts. However, there are various other reading strategies to consider. The first reading could be more cursory, while subsequent readings—perhaps after conducting an initial pilot experiment with an eye tracker, or after consulting the other parts in the article series—might focus more on the finer details. The article can also serve as a reference, allowing readers to focus on specific sections of interest. Given that this article can be approached in different ways, we have chosen to use jargon commonly found in the scientific literature without always providing explanations. However, we have included references when introducing key concepts, enabling the interested reader to explore them in greater depth if desired.

## About operationalizations

According to Emmerich et al. (2016): “operationalization is a substantial aspect of quantitative research and generally referred to as the process of defining how to quantify a phenomenon or concept which itself is not directly measurable”. It is clear that operationalization of attention is necessary because its existence is inferred from other phenomena (e.g., by fixation duration, fixation position, saccade curvature, and manual reaction time). Seemingly measurable concepts are often also not directly measurable. For example, pupil size and gaze direction may appear directly measurable but are not. They are indirectly estimated from the recorded eye image using algorithms. Some classes of concepts are more contentiously operationalized than others. By contentiously, we mean that there is discussion or less disagreement about it in the literature. For example, the use of the saccade detection algorithms of Komogortsev et al. (2010) is less controversial than the application of the eye-mind hypothesis of Just and Carpenter (1976). This is to be expected because for most researchers the concept of a saccade is less fuzzy than the concept of attention.

When researchers reach a consensus on a specific operationalization, it becomes possible to compare empirical results across different studies and have fruitful discussions. Agreement about operationalizations is helpful. However, it also has a drawback. If a part of a research field ceases to engage in discussions about a particular operationalization, it may give the impression to newcomers in the field that the operationalization has replaced the theoretical concept. Consequently, there might be a lack of critical examination regarding the nature of the operationalization and the underlying assumptions. A notable example is the consideration of the pupil-size signal derived from an eye tracker as the actual size of the pupil, which is incorrect for most eye trackers, because the pupil appears smaller in the eye image when the eye is in an eccentric viewing orientation (Hayes & Petrov, 2016).

The present article delves into the operationalization of research questions related to the movements of the eye or by the use of movements of the eye. While certain studies, particularly those describing basic eye movement patterns, lend themselves to straightforward operationalization (i.e., record the eye movements during the appropriate conditions and report relevant parameters such as saccade amplitude, number of fixations or fixation time), complexities arise in research fields where eye movements serve as proxies or probes for processes such as perception, attention, or cognition. The latter is considerably more complicated; eye movement metrics such as fixation duration, fixation location, saccadic peak velocity or saccade curvature are now regarded as proxies for concepts that are not directly measurable. This is not without problems and many operationalizations come with discussions and assumptions.

### Direct and indirect operationalization

Seemingly measurable concepts can often be operationalized directly by something that has a strong (and often quantitative) relation with the original concept. The operationalization of concepts regarding the nature of eye movements is relatively straightforward because it concerns direct operationalization. Illustrating this with an example, Bahill et al. (1975b) aimed to explore the relationship between peak velocity and the amplitude of saccades. While some knowledge existed on this relationship, Bahill et al. (1975b) sought to investigate it across a broader range of saccade amplitudes. The experimentation involved recording an ample number of saccades spanning a wide amplitude range and determining their peak velocity. The operationalization only involved saccade amplitude, saccade duration, and saccade peak velocity. Other examples of topics involving direct operationalization are the pupil reaction to light (Reeves, 1920), the description of blinks (Blount, 1927), the size of the oculomotor range (Collewijn et al., 1988a) and a mathematical description of 3D eye orientation and velocity (Tweed & Vilis, 1990). Closely tied to accurately describing eye movements is understanding the control of these eye movements. Examples include saccade generation (Robinson, 1973, 2022; Bahill et al., 1975a; Bahill & Stark, 1979), models for comprehending combined eye-head movements (Bartz, 1966; Freedman, 2008) and the control of 3D eye orientation (Angelaki & Hess, 2004). The operationalization of concepts in these studies is also often straightforward and involves accurate and precise measurement of eye movements. It is important to note that we do not claim simplicity in studying the nature and control of eye movements. To investigate and accurately describe these movements, high-quality eye trackers and gaze estimation methods are necessary (e.g., Collewijn et al., 1975; Collewijn et al., 1985; Crane and Steele, 1985; Robinson, 1963). Researchers in this field have invested significant time and effort in developing new eye trackers, gaze estimation methods, calibration methods (Poletti & Rucci, 2016; Drewes et al., 2014; Stampe, 1993), sophisticated mathematical models (Raphan, 1998) and eye-tracking data processing methods (Van Rijn & Van den Berg, 1993). Examples include the study of saccades (van der Steen & Bruno, 1995; Collewijn et al., 1988b), vergence (Zee et al., 1992), pupil movements (Hooge et al., 2015), microsaccades (Steinman et al., 1973; Engbert, 2006; Nyström et al., 2017; Ko et al., 2010), and smooth pursuit (Robinson, 1965; Goettker & Gegenfurtner, 2021).

The story of what we refer to as indirect operationalization is more complicated. In the case of indirect operationalization, one substitutes the concept with something that exhibits an observable relationship with the intended concept. For instance, if we are interested in studying cheerfulness, we may opt to investigate laughter or smiling. Laughter or smiling can then be operationalized by the observable transformation of facial muscles, the exposure of teeth, and the narrowing of the eyes, for example. The same principle can be applied to operationalizations in eye-movement research. The concept “having interest in a person” can be substituted by a more overt focus on a person. Attention is a complex notion, but overt attention can be operationalized as having prolonged gaze towards a person. Alternative operationalizations are feasible as well; for instance, placing the person among other people, thereby operationalizing interest in the person through relative gaze measures (prolonged gaze to one person compared to other people). It should be evident that empirical outcomes may vary depending on the chosen operationalization.

## Operationalizations of many common concepts in eye tracking

In eye-tracking research, various operationalizations are employed for numerous concepts. These include quantifying the number of black pixels in the eye image of an eye tracker as an estimate of pupil size, as well as utilizing areas of interest to determine attention towards specific visual elements in an image. While some operationalizations, such as the fixation and saccade events by the SR Research Data Viewer^1^, are hardly controversial, while others prompt discussion. An illustrative example of such a debate revolves around whether the fixation location can be considered as the locus of visual attention. Rayner (2009a, p. 1458) writes: “It is my contention that most of the time in such tasks, either (a) eye location (overt attention) and covert attention are overlapping and at the same location or (b) attention disengagement is a product of a saccade program (wherein attention precedes the eyes to the next saccade target)”. Regardless of whether the operationalization is debated, we emphasize the importance of alerting researchers to the assumptions associated with most operationalizations. Researchers should be conscious of the (hidden) assumptions they (implicitly) adopt when selecting a particular operationalization. Too many assumptions in the operationalizations might undermine the generalizability of the conclusion of a study.

The choice of an operationalization may also have implications for the estimated values of a concept; for instance, blink durations determined from the eye openness signal are generally longer than those derived from the pupil signal (Nyström et al., 2024). Some implementations of operationalizations are not fixed; changing parameters of the fixation detector in Tobii Pro Lab^2^ may alter the fixation duration and the number of fixations. Not being transparent about the details of the implementation of the operationalization (e.g., the settings of a fixation classifier) can hinder the interpretation of eye-tracking data or comparison of results across different studies (cf. Carter and Luke, 2020; Dunn et al., 2024; McConkie, 1981; Oakes, 2010). In case that the estimated fixation durations are very short compared to the fixation durations reported in the literature, it may be necessary to adjust the selection rules of the fixation classification (Hooge et al., 2022). An example of a selection rule from reading research is the recommendation by Inhoff and Radach (1998) to exclude fixations with durations below 50 ms, “assuming that shorter duration fixations are not determined by on-line cognitive processes” (p. 34).

The following section provides a non-exhaustive list of operationalizations for key concepts of eye tracking. The concepts are ordered from seemingly measurable and directly operationalized (fixation) to indirectly operationalized (mind-wandering).

**Fig. 1:**
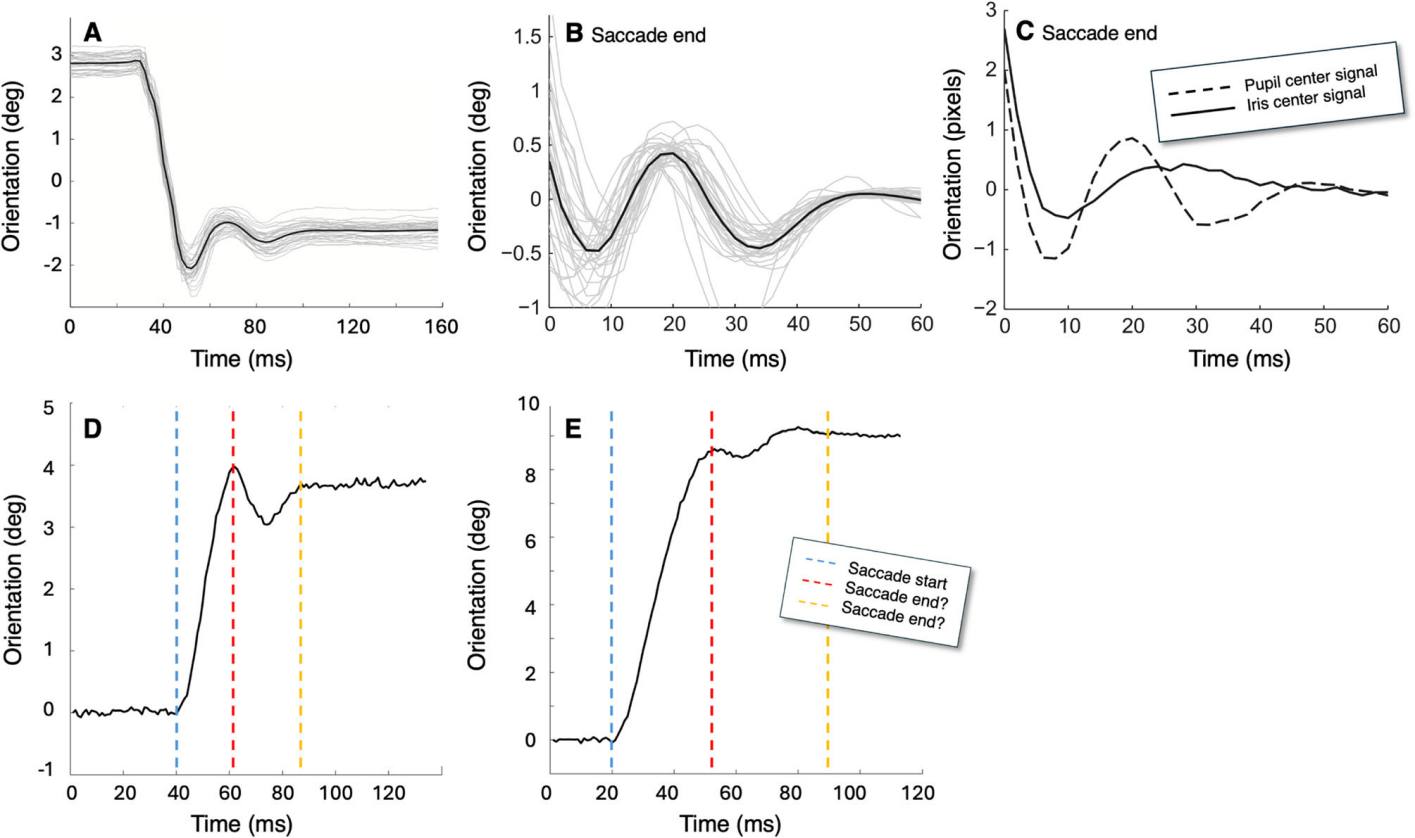
Saccade with post-saccadic oscillation (PSO). Panel **A** shows a saccade waveform of a 4$^\circ$ saccade (adapted from Hooge et al., 2015). The *thick black line* is the average of 34 saccades (the *thin grey lines*). The PSO can be seen at the end of the saccade. It is not clear at what point in time the saccade ends and the PSO begins, as they likely overlap. Panel **B** (adapted from Nyström et al., 2013) zooms in on the PSO in the eye tracker signal (from a different saccade than in panel A). Panel **C** (adapted from Nyström et al., 2013) shows the movement of the pupil and the iris center from the PSO depicted in panel B. From panel C, it is clear that the iris center (and thus the entire eye) oscillates less and slower than the pupil center. The eye tracker signal from panel B correlates highly with the pupil center signal of panel C, suggesting that the PSO in the eye tracker signal represents pupil center movement (not eyeball rotation). Panels **D** and **E** (adapted from Hessels et al., 2018) depict a 4$^\circ$ and a 9$^\circ$ (undershoot) saccade. The *blue dashed line* depicts the saccade start. As the saccade end and PSO overlap, the *dashed red* and the *yellow lines* depict two suggestions for the many possible saccade ends, illustrating the problem that the occurrence of PSOs raise for the determination of the saccade end

### Fixations and saccades

When conducting research using an eye tracker, one may need operationalizations of fixations and eye movements (e.g., saccade, vergence, smooth pursuit, cyclovergence). Despite appearing to be a straightforward and long-solved issue, terms such as fixation and saccade are not always clearly defined (Hessels et al., 2018). The theoretical description often lacks specificity compared to the algorithmic operationalization. An example of an algorithmic operationalization is provided by Cornelissen et al. (2005): “Saccades were detected off-line using a velocity criterion of 30$^\circ$ $/$s, an acceleration criterion of 8500$^\circ$ $/$s$^2$ , and a displacement criterion of 1.0$^\circ$”. Conversely, algorithms may be prone to errors and susceptible to noise, artifacts, and systematic phenomena. An example of such a systematic phenomenon is the post-saccadic oscillation (PSO, Hooge et al., 2015; Nyström et al., 2016; Hooge et al., 2016; Nyström and Holmqvist, 2010; Nyström et al., 2013). During and after a saccade, the inner-iris border (the pupil) undergoes movement relative to the rest of the iris (see Fig. 1C). Many video-based eye trackers utilize the pupil center to estimate gaze. Specifically, following a saccade, the pupil oscillates in relation to the entire eyeball. This oscillation, observable in sufficiently sensitive eye tracker signals, starts before the end of a saccade and continues until after the end of the saccade. This poses a challenge (see Fig. 1D and E) in estimating the exact end of the saccade and determining its duration (Hooge et al., 2015). There is no clear-cut solution for determining the end of the saccade in the signal from a pupil-CR eye tracker. However, Nyström and Holmqvist (2010) proposed a suggestion in their algorithm, in the form of a PSO classifier. At the very least, they prevent the period of the PSO from being included in the saccade duration at the risk of underestimating the duration of the saccade.

Factors influencing the choice of saccade and fixation detectors include precision in eye-tracking data, data loss, and recording frequency. Various fixation and saccade detectors exist (Nyström & Holmqvist, 2010; Komogortsev et al., 2010; Engbert & Kliegl, 2003; Smeets & Hooge, 2003; Hooge & Camps, 2013; Hessels et al., 2017; Startsev et al., 2019). Is one better than the other? Andersson et al. (2017) evaluated different algorithms for saccade and fixation detection against human coders. Depending on the nature of the eye-tracking signal (e.g., the absence or presence of smooth pursuit episodes), some algorithms outperformed others. Hooge et al. (2022) focused on fixation classification, asserting that the specific algorithm choice is of minimal importance, provided it is sensitive enough, and appropriate values for minimal fixation duration (>60 ms) and minimal saccade amplitude (>1$^\circ$) are chosen. For a recent review on eye movement event detection, we recommend Startsev and Zemblys (2023).

Many researchers may not realize they have the option to choose an operationalization for fixation, saccade, and blink detection. Often, they employ corporate software (e.g., Tobii Pro Lab, SR Research Data Viewer). These packages include algorithms with effective default settings for blink, saccade, and fixation classification. The advantage of corporate software lies in its tailoring of operationalizations to the eye tracker data’s characteristics. An example of the previous is the saccade and fixation detector in SR Research Data Viewer. However, we recommend users of corporate software to utilize the visualization features to verify whether the classified fixations and saccades align with their intuitive understanding or expectation of these phenomena. We especially recommend novices to look at the raw eye-tracking signal to familiarize themselves with it.

In the literature, numerous open-source and free software options are available for extracting various eye movements from eye-tracking signals. Specialized software packages address specific issues, such as microsaccade detection (Mihali et al., 2017), or fixation classification in infant eye-tracking data (Hessels et al., 2017). This article does not delve further into this topic, deferring to Niehorster et al. (2025) for a comprehensive description of tools researchers can use for their eye-tracking studies.

The operationalization of fixations and saccades is often algorithmic. Researchers are sometimes not even aware that the settings of an algorithm may play an important role in the operationalization. Agreement about an operationalization may involve using the same algorithm with the same settings. Transparency about the algorithms and the settings used is therefore important when comparing results from different studies.

### Pupil size

Pupil size (Loewenfeld, 1999) serves as a prevalent operationalization in the assessment of various cognitive states (Strauch et al., 2022; Papesh & Goldinger, 2024), including workload (Pfleging et al., 2016; Hess, 1965), attention (Mathôt et al., 2013; Binda et al., 2013) mental fatigue (Bafna & Hansen, 2021), and arousal (Bradshaw, 1967; Bradley et al., 2008; Wang et al., 2018). Many video-based eye trackers produce a pupil-size signal. It is important to note that these eye trackers do not measure pupil size directly. The operationalization of pupil size may involve quantifying the number of black pixels within the image of the pupil or determining the surface area of an ellipse fitted to the image of the pupil. However, a significant challenge associated with this operationalization arises from the dependency of both pixel count and pupil area on the orientation of the eye axis relative to the eye camera of the eye tracker (Hayes & Petrov, 2016; Gagl et al., 2011) and the refraction of the cornea (Petersch & Dierkes, 2022). Imagine a participant with a fixed pupil size. When this participant looks to the right, the pupil in the camera image appears smaller due to the perspective compared to looking straight ahead. Tobii claims that their eye trackers report physical pupil size. According to Tobii Pro AB (2024): “Pupil size is calculated by measuring the pupil’s diameter in the image and multiplying it by a scaling factor”. How effective is Tobii’s method? Brisson et al. (2013) concluded that for the Tobii eye trackers they tested (Tobii T120, Tobii X120), there was insufficient correction applied. Researchers may opt to employ the methods proposed by Gagl et al. (2011) or Hayes and Petrov (2016) to correct for gaze angle when assessing pupil data. Another approach involves designing the study to minimize eye movements or eccentric viewing angles. Toet et al. (2017) instructed participants to fixate on a single point during the period that the pupil size was estimated, thereby mitigating issues related to distortion and constriction of the pupil due to perspective effects. For recent methodological reviews we recommend Fink et al. (2024); Mathôt and Vilotijević (2023) and Steinhauer et al. (2022).

### Blink

In addition to eye movements and pupil size, an eye tracker can be utilized to determine instances of blinks (closing and re-opening of the eyelids), both incomplete and full blinks (see Table 1 in Nyström et al., 2024, for a recent review). Blink rate serves as a potential metric for assessing fatigue (Stern et al., 1994), workload (Stern & Skelly, 1984), and is associated with cognitive processes (Eckstein et al., 2017). Most methods accompanying a video eye tracker detect blinks based on the pupil-size signal. Specifically, blink events are operationalized as periods of data loss that are neither too long nor too short. A very long period of data loss may be caused by not looking at the screen and a very short period of data loss may be caused by something else (e.g., a brief tracking problem associated with, e.g., downward-pointing eyelashes). Another method to detect blinks involves utilizing the eye openness signal (Nyström et al., 2024).

**Fig. 2:**
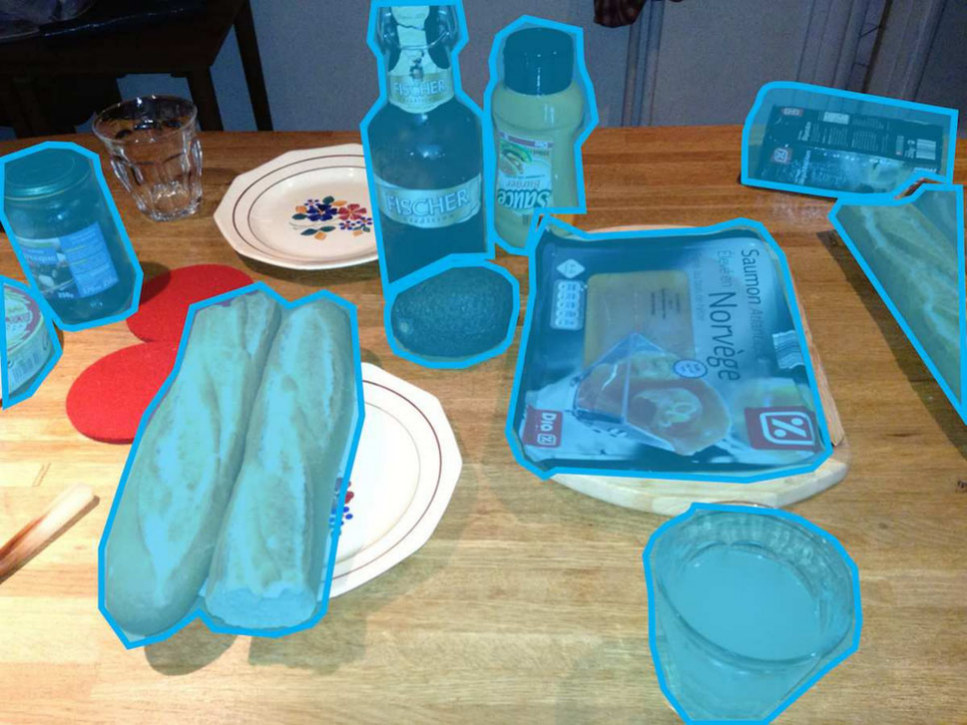
An example of food AOIs. In this illustration, so-called non-overlapping AOIs are depicted with a rather strict delineation. This choice was made by the creator of these AOIs. The authors of this article do not take a stance for or against the depicted AOIs. These AOIs have both advantages and disadvantages. A fixation outside the AOI boundaries, for instance, due to imprecise or inaccurate eye-tracking, is not included in the AOI count (miss). Similarly, a fixation adjacent to an AOI because the viewer was not looking at the AOI is not considered an AOI hit (correct rejection). This AOI set has a lower probability of producing false alarms and a higher probability of misses than an AOI set with boundaries drawn more generously around the objects

### Spatial looking behavior

In many eye-tracking studies, the focus lies in describing aspects of spatial gaze behavior. Where did the participants look? At times, the researcher may solely seek to ascertain whether the participants looked towards the left or right on the screen (e.g., Shimojo et al., 2003) or at the dials in the cockpit (e.g., Allsop and Gray, 2014). Researchers in these studies are frequently concerned with identifying the objects within an image or a movie clip that are looked at, rather than delving into the pixel coordinates as a function of time. In such a scenario, one can use areas of interest (Salvucci & Goldberg, 2000; Krugman et al., 1994; Janik et al., 1978). An area of interest (AOI) is a region within the visual stimulus (see Fig. 2) that the researcher intends to examine for the corresponding eye-tracking data (Holmqvist et al., 2011). Possible inquiries include the mean duration of participants’ gaze within the AOI, the frequency of revisits, and the total time spent looking at the AOI. An illustrative example is a study by Pieters and Wedel (2004) from marketing research. They were interested in estimating attention capture and message transfer for magazine advertisements. To do this, they used AOIs for visual elements, brand and text of the advertisements and used these AOIs to quantify various aspects of spatial looking behavior. Another example is the investigation in the field of cartography conducted by Franke and Schweikart (2017), exploring the efficacy of representing landmarks on abstract maps as vignettes, pictograms, or text symbols.

By the use of AOIs, many useful statistics can be conducted (e.g., time to first AOI hit, time spend in an AOI, average AOI visit time Holmqvist et al., 2011, Chapter 6, p. 187). From AOI measures, higher-order descriptives can be computed. One may think of a scanpath analysis, a useful method to study the order of looking (Cristino et al., 2010; Eraslan et al., 2016; Dewhurst et al., 2012; Hooge & Camps, 2013; Goldberg & Helfman, 2010). Another higher-order descriptive is the transition matrix (Goldberg & Kotval, 1999), describing the frequency of the transitions between AOIs. Based on transition matrices, researchers may also develop more complex models to describe looking behavior (e.g., Kucharský et al., 2020). Researchers interested in the degree of orderliness of gaze behavior may compute various entropy measures based on the scanpaths or transition matrices (Allsop & Gray, 2014; Hooge & Camps, 2013; Niehorster et al., 2019; Krejtz et al., 2014; Kruizinga et al., 2006).

Various types of AOIs exist and for an exhaustive account of them (e.g., semantic, gridded, dynamic, whitespace, distributed, fuzzy), we refer to Holmqvist et al. (2011, Chapter 6, p. 187). It is crucial to emphasize that the creation of AOIs is a specialized endeavor that is often underestimated. The appearance of an AOI, as an operationalization of a specific meaningful part of a visual stimulus (e.g., nose, tree, tool) may significantly impact the conclusions of a study. The shape and size of an AOI may affect the statistics (Holmqvist et al., 2012). If the AOI is too small, fixations may be missed, if the AOI is too large, fixations to other objects may be counted (see Fig. 2). Orquin et al. (2016) write: “Ideally, an AOI includes all fixations belonging to an object while fixations to other objects are excluded”. One of the problems is, how does a researcher know whether the AOI size is appropriate for the research question? Our answer is that unless the researchers conducts psychophysical experiments to find out how far an observer can fixate next to an object and still see or identify that object, the researcher does not know. Sensible advice to draw AOIs is given in Hessels et al. (2016). If the stimulus is sparse, one should make the AOI as big as possible. The reason is that fixations are usually clustered around the few informative parts of the sparse display. Usually, people do not fixate very often in “empty” parts.

Consider a scenario where a researcher aims to create AOIs for each person appearing in a TV commercial; should these AOIs be dynamic (i.e., should the AOI follow the moving person)? This depends on the nature of the individuals in the TV commercial. If individuals move from left to right, the use of dynamically moving AOIs is necessary. When there are almost no significant movements in the movie clip, static AOIs can also be utilized. The following example concerns a less dynamic movie clip and static AOIs sufficed. Wolff et al. (2015) employed a (static) gridded AOI to delineate gaze behavior disparities between novice and expert teachers while watching a movie clip of a classroom. The gridded AOI, which constitutes dividing the image area in *n* equally sized rectangular parts (introduced by Buswell, 1935) is not aimed at specific objects but rather at regions within the image frame. Gridded AOIs are only useful if the “what” in terms of semantics is less relevant.

Many researchers utilizing eye trackers tend to associate gaze positions with the objects in a visual stimulus and opt for an AOI analysis. In some instances, the AOI approach can be complex, labor-intensive, and sometimes unnecessary. One of the problems with AOI production is how to produce AOIs in a reproducible and objective way. Reproducibility is important, for instance, when we aim to replicate findings. One solution is to use image processing algorithms to produce static (e.g., Fuhl et al., 2018; Hessels et al., 2016) or dynamic AOIs (e.g., Hessels et al., 2018; Hessels et al., 2020; Körner et al., 2024; Wolf et al., 2018). Not all problems lend themselves for automatic AOI production. However, some visual stimuli are particularly suitable for the automatic generation of AOIs, such as text displays (the option for automatically determining word boundaries is present in many corporate software suites), computer-generated search displays (Hooge & Erkelens, 1999), and augmented and virtual reality environments.

It is not always required to map gaze to objects or regions in the stimulus to answer a research question concerning spatial behavior. When the question is not about the “what in the world is looked at” but instead about “how often or how far gaze is shifted in the world”, an AOI analysis is not required. For example, Dowiasch et al. (2015) investigated the impact of aging on eye movements in real-world settings by assessing saccade frequency, saccade amplitude, peak velocity, and mean velocity during walking in a corridor. Another example can be found in Antes (1974), Over et al. (2007) and Unema et al. (2005). They explored the spatial and temporal aspects of fixation duration and saccade amplitude during scene viewing and visual search and reported coarse to fine scanning behavior. Participants start with large saccades and short fixation times, and, as a function of time saccade amplitude decreases and fixation time increases. Researchers can save considerable time and effort if mapping gaze to the visual stimulus is not required for answering their research question.

### The three-dimensional fixation point

Where do people look (attend) in the (virtual) world? This is an interesting question for many (applied) researchers. Stein et al. (2022) investigated how individuals with varying expertise observed two different sculptures. The participants were allowed to walk around the sculptures and could view the sculptures from different viewpoints. Gaze behavior was analyzed using eight reference images taken from different directions. This implies that they approached gaze behavior as a two-dimensional problem. In research using this type of analysis, the point of regard is operationalized as the intersection of the line of sight with an object (such as the screen of a remote eye tracker).

Can it also be approached as a three-dimensional problem? In other words, can the location of the fixation point in 3D be estimated with a binocular pupil-based video eye tracker? The intersection of the two individual lines of sight from the two eyes is termed the binocular fixation point (Collewijn et al., 1997). For illustrations of the three-dimensional fixation point see Nuthmann and Kliegl (2009, Fig. 2) or Hooge et al. (2019, Fig. A2). The angle between the lines of sight is called the vergence angle. Wismeijer et al. (2010) used a binocular video-based pupil-CR eye tracker to study what visual information controls vergence. They found that vergence was dominated by one source of information, namely binocular disparity. Gibaldi and Banks (2019) studied the relation between the natural-disparity distribution and vergence. They report that vergence was quite consistent with the natural-disparity distribution.

If an eye tracker is sufficiently accurate, the three-dimensional fixation point can be estimated based on the vergence angle. The challenge with modern pupil-CR video-based eye trackers lies in their inability to accurately estimate gaze direction due to the pupil size artefact (PSA, Choe et al., 2016; Drewes et al., 2014; Jaschinski, 2016; Wyatt, 2010). What is the PSA? The majority of human inner iris borders (pupils) do not constrict around a fixed point with respect to the eyeball. This implies that when the pupil constricts, the center of the pupil shifts without a corresponding change in the observer’s gaze direction. The eye tracker’s algorithm uses the pupil center to estimate gaze direction. Consequently, when the pupil constricts, apparent gaze shifts may occur, making gaze estimation inaccurate. The vergence angle represents the disparity between the left eye’s viewing direction and the right eye’s viewing direction, and a minor inaccuracy in either eye orientation can lead to a substantial shift in the estimated binocular fixation point in space (see Fig. 1 in Hooge et al., 2019). However, a compensation for inaccuracy due to PSA has been proposed (Drewes et al., 2014).

A pupil-based video eye tracker is not the ideal eye tracker for determining the position of a three-dimensional fixation point. More accurate eye trackers, such as the dual-Purkinje-image eye tracker (DPI, Crane and Steele, 1985) or scleral coils (Collewijn et al., 1975), are better suited for this purpose. However, when a researcher wants to know which object in the environment is being looked at, a pupil-based video eye tracker can be adequate under certain conditions (e.g., with two-dimensional methods, in sparse environments, orclose to the observer (Mlot et al., 2016; Hooge et al., 2019))

### Eye contact

Eye contact has been a widespread topic of study in the 60s to 80s of the last century (e.g., Argyle and Dean, 1965; Kendon, 1967; Kleinke, 1986; Rutter et al., 1984). However, in most of these earlier studies, eye contact was assessed through, e.g., direct observation or by annotation of video recordings, not with eye trackers. A notable exception is the study of Haith et al. (1977), who used CR eye tracking to investigate eye contact in early infancy. With the advance of modern remote and wearable eye trackers, eye contact is now studied in much more varied contexts (e.g., Auyeung et al., 2015; Franchak et al., 2018; Hessels et al., 2018; Honma, 2013; Niedźwiecka et al., 2018; Ye et al., 2012). Given the long history of studies on eye contact (see e.g., Hessels, 2020; Hietanen, 2018; Kleinke, 1986; Senju and Johnson, 2009), there are many different operationalizations. For example, eye contact can be operationalized as the gaze point being located in the eye or the face region of another person, but also as episodes where it feels like eye contact is being made. For a recent review on eye contact and its various operationalizations, see Jongerius et al. (2020).

### Overt attention

Attention is a complex concept (Hommel et al., 2019). The term has many definitions but also meanings in daily use. In eye-tracking research, we observe that gaze behavior, overt attention, and attention are often used interchangeably. In more explicit cases, overt attention is operationalized by eye gaze (Beesley & Le Pelley, 2011), or an overt attention shift refers to the employment of head and eye movements to gaze at an item (De Haan et al., 2008). Given the many definitions, there are also many possible operationalizations. Here, we specifically discuss two aspects of overt attention.

#### Attention-attracting power

Eye tracking is a popular tool to investigate media content (e.g., websites, social media, magazines, outdoor advertisements). People are bombarded with visual information, and for ads to be effective, they need to cut through the clutter of competing advertisements and editorial content (Pieters & Wedel, 2004). Attention-attracting power is the ability for an ad or a part of an ad to capture overt attention (e.g., looks, glances, and fixations). Suppose one investigates the attention-attracting power of visual elements (e.g., text or brand expressions) in outdoor advertising by having 50 participants view an image of outdoor advertising on a computer monitor and record their gaze. What constitutes the most effective attention magnet on the outdoor advertisement? One may think of different possible operationalizations. The simplest operationalization we can think of is the highest peak in a heatmap representation of gaze data (Špakov & Miniotas, 2007). However, most heat maps do not contain temporal information.^3^ The highest peak signifies the location where fixations most frequently occur, and it is plausible that individual observers may revisit a particular location for a second or third time. The heat map also does not indicate which element was fixated on early. The same applies if we take the most visited AOI as the attention magnet.

The most effective attention magnet could also be operationalized as the visual element that is viewed most rapidly for the first time by the majority of observers. However, the terms *rapidly* and *majority* introduce a challenge. Consider one AOI, which is looked at by 22 out of 50 participants, and they do so with an average latency of 1.33 s. Another AOI is viewed by 12 out of 50 participants in an average time of only 0.7 s. Hooge and Camps (2013) proposed a solution to this dilemma. For each AOI, it is possible to calculate the time elapsed before 50% of participants have looked for the first time at the AOI, known as the T_50_. The AOI with the shortest T_50_ is considered the most effective attention magnet. If not enough participants fixated the AOI, one may also consider computing T_40_ or T_25_.

#### Attention-retaining power

In addition to attracting attention, retaining attention is also crucial in, e.g., marketing and media research. A visual element must be observed for a sufficient duration to effectively convey its message to the viewer. The capacity to retain attention can be operationalized by the time spent looking in the AOI. There are various labels for this measure including looking time (Falck-Ytter et al., 2013), dwell time (Holmqvist et al., 2011), total fixation time (Lai et al., 2013) and glance duration (Zhang et al., 2006). A look (or dwell or glance or visit) may comprise multiple fixations. Moreover, definitions of these terms may differ substantially. For definitions, see Holmqvist et al. (2011, p. 190) and Attardo and Pickering (2023, p. 62). One very explicit example is given in ISO (2014), according to which a glance includes the time of the saccade leading to the first fixation position within the AOI and then all the fixations and saccades within this AOI. This operationalization is in the context of how much time people spend looking at the road or their dashboard.

### Gaze cueing, guidance, following, and joint attention

A substantial amount of research has been conducted on the relation between one’s gaze and that of another person (see e.g., Birmingham and Kingstone, 2009; Emery, 2000; Frischen et al., 2007; Hessels, 2020; Shepherd, 2010). Consequently, there are multiple ways in which that relation can be expressed. For example, the gaze direction of another person may cue one’s spatial (covert) attention automatically. This is often operationalized in a gaze cueing experiment as shorter manual or saccadic reaction times to a target appearing in the gazed-at (cued) location versus the non-gaze-at (uncued) location (e.g., Friesen and Kingstone, 1998). Whether cueing by means of another person’s eyes is different from, e.g., biologically less relevant stimuli (arrows) has been a topic of debate (e.g., Ristic et al., 2002).

Other ways of operationalizing the relation between one’s gaze behavior and the gaze direction of another person include gaze guidance and gaze following. Gaze guidance may refer to the fact that an object, for example, a Rolex watch in an ad, is looked at more or longer when a person depicted in that ad looks in the watch’s direction (Hutton & Nolte, 2011). Gaze following may refer to the frequency of making a gaze shift in the same direction or towards the same object that another person just made a gaze shift towards (Senju & Csibra, 2008; Thorup et al., 2016; Hernik & Broesch, 2019).

A related concept is that of joint attention, which may refer to periods when two people look at the same object at the same time. Practically, this may occur following successful gaze following as described above, but may also occur in the context of e.g., parent–infant interaction without a previous gaze-following episode (Yu & Smith, 2013). Specific operationalizations may include the number of concurrent object-fixations by two or more people, or the average and total time of such occurrences. There are also taxonomies for distinct phases (e.g., initiating or responding to joint attention) leading up to joint attention in the context of human interactions (Jording et al., 2018). The research field on gaze behavior in interaction with other people is extensive, and interested readers are referred to review articles on this topic in the context of eye-tracking research (e.g., Hessels, 2020; Pfeiffer et al., 2013; Valtakari et al., 2021).

### Appreciation of visual elements

An intuitive assumption is that people gaze longer at things they appreciate. Leder et al. (2016) observed that more and longer fixations occur on aesthetically pleasing individuals. Pelowski et al. (2018) articulated: “We expected that longer fixation times on the artworks (as opposed to other elements of the rooms such as floors and ceilings) would coincide with more positive or emotional experiences”. Appreciation could then be operationalized through longer fixation time, more fixations, or a combination of fixation time and number of fixations that signify an extended viewing duration on the respective object. However, it is not that straightforward. Individuals also gaze longer at objects when the visual task is more challenging. Difficulty can mean lower visibility (e.g., due to lower contrast, more elements, and increased clutter) or harder comprehension (for a review of fixation times in scene viewing, see Nuthmann, 2017). This implies that longer fixation durations do not necessarily indicate that the viewer has greater appreciation for the object or person looked at. Brieber et al. (2014) conducted a study highlighting the complexity of this topic. Higher-rated paintings were observed for a longer duration. They also discovered that paintings rated more ambiguously and with a higher understanding rating^4^ were looked at longer. To further complicate matters, the context (lab or museum) also played a role in these relationships. Appreciation can, therefore, not simply be operationalized as longer viewing time. Concepts such as appreciation, interest, engagement, novelty (non-exhaustive list) are probably more easily operationalized by a simple questionnaire.

### Covert attention

Let us begin with a theory explaining why it is advantageous for individuals to possess covert attention. Humans exhibit a white sclera adjacent to a darker-colored iris, making it possible for an observer to discern where one’s overt attention is directed. Laidlaw et al. (2016) state: “Covert visual attention - that is, attending without a related eye movement - serves to bias perceptual and neural processes of salient or behaviorally relevant stimuli”, which can be particularly useful in social situations when one does not want to overtly attend another person.

Consider a scenario in which a researcher aims to investigate, using eye tracking, the locus of covert attention and its temporal dynamics. How can covert attention be operationalized with an eye tracker? This is of course more complex than operationalizing overt attention. The latter can be operationalized directly with the point of regard. Duchowski (2002) writes about covert attention: “The dissociation of the “spotlight of attention” (Posner, Snyder, & Davidson, 1980) from ocular fixation poses a problem for eye-tracking researchers”. However, several experimental techniques are employed for operationalizing covert attention. These techniques all have in common that they are indirect. The first technique is based on the work of Posner and Cohen (1984), specifically cueing effects involving inhibition of return (IOR). This phenomenon describes the effect whereby locations previously attended to (both overtly and covertly) exhibit a delayed ability to be fixated upon again (Hooge et al., 2005; Klein, 2000; Lupiáñez et al., 2006; Lupiáñez, 2010; Rafal et al., 1994). Also based on the work of Posner and Cohen (1984) is that saccades to a cued location have shorter latency. The second phenomenon to be considered is that of curved saccades, which either deviate away from or towards locations where covert attention was (previously) directed at (Van der Stigchel & Theeuwes, 2007; Doyle & Walker, 2001; Godijn & Theeuwes, 2002; McPeek et al., 2003; McSorley et al., 2006). A third method to operationalize covert attention is through the use of microsaccades. Hafed and Clark (2002) and Engbert and Kliegl (2003) demonstrated that the rate and direction of microsaccades change depending on a cue’s onset and location. The last phenomenon we want to describe demonstrates higher pre-saccade detection accuracy at locations programmed for saccadic movements (e.g., Deubel and Schneider, 1996) suggesting that the locus of covert attention precedes the saccade. Thus, we find at least four potential operationalizations of covert attention; 1) inhibition of return and cueing effects, 2) saccade curvature, 3) micro-saccade rate and micro-saccade direction, and 4) pre-saccadic facilitation.

The primary reason perhaps to utilize an eye tracker in covert attention research is not to operationalize covert attention. An eye tracker can be employed to verify whether the participants adequately fixate on the central fixation point to make sure that covert attention is studied (Spence & Driver, 1997, 1998; McDonald et al., 2003). One could go even further and utilize gaze-contingent displays to ensure that if participants do not accurately fixate on the central fixation point, the peripheral visual stimulus disappears.

### Workload

According to Cain (2007): “Despite interest in the topic for the past 40 years, there is no clearly defined, universally accepted definition of workload”. Workload is often approached with multiple sensors and methods (e.g., physiological, cardiovascular, eye movement, EEG, respiration, skin, EMG, and neuroendocrine measures, Tao et al., 2019). Eye tracking is used to estimate workload in various work fields and for different topics, including air traffic control (Ahlstrom & Friedman-Berg, 2006), visual search (Backs & Walrath, 1992), artificial intelligence support (Buettner, 2013), cognitive tasks (Granholm et al., 1996), driving (Marquart et al., 2015), an anesthesia simulator (Schulz et al., 2011), during a free-viewing task (Tokuda et al., 2011), the n-back task (Hogervorst et al., 2014), surgery (Tolvanen et al., 2022), auditory and driving tasks (Tsai et al., 2007) and a visuospatial memory task (Van Orden et al., 2001).

Lysaght et al. (1989, p. 146) wrote about eye tracking that: “The cost and effort required to obtain and analyze eye movement data reduce the practical applicability of these techniques”. About pupil size measurements they wrote: “Pupil diameter has been shown to be sensitive to workload variations, especially the amount of mental load. However, measurement techniques do not lend themselves to field situations. These restrictions limit the technique to the laboratory”. Nowadays, this is outdated, because of the miniaturization of eye trackers. Since the 2010s, there are good wearable eye trackers available that can record both pupil size and eye movements outside the lab (e.g., Fotios et al., 2015a, b). Outside the lab is not necessarily outdoors, where variation in light conditions makes it difficult to study the relatively smaller pupil size changes due to, for instance, workload. It can be, e.g., in a simulator, a classroom, a submarine or an office. Most modern wearable eye trackers are robust for slippage (Hooge et al., 2023; Niehorster et al., 2020).

In a recent literature review, Tao et al. (2019) showed that the metrics used in estimating workload include blink rate, pupil diameter, blink duration, fixation duration, saccade velocity, fixation rate, saccade rate, saccadic amplitude, blink amplitude, blink interval, fixation spread, saccade duration, and dwell time. Most studies reported significant differences in blink rate, pupil diameter, and fixation duration across varying mental workload levels. High visual workload decreased blink rate in air traffic control and flight simulation tasks. Pupil diameter increased in demanding tasks like air traffic control and nuclear power plant operations. Fixation duration decreased with higher task demands in simulated flight and driving tasks. Blink duration generally decreased in high-complexity tasks. Saccade-related measures (velocity, rate, amplitude, duration) were mainly reported in aviation, showing decreased values with increased cognitive load. Other measures like fixation spread and dwell time had limited validation.

We discuss three methodological issues with pupil size, blink rate, and saccade velocity. Pupil size varies not only as a function of workload but also with changes in light intensity. It is claimed that the index of cognitive activity (ICA) successfully separates the light reflex from the pupil dilation due to cognitive effort (Marshall, 2002). Bartels and Marshall (2012) tested the usability of the pupil size signal from several eye trackers to determine the ICA. All tested eye trackers (recording frequency ranging from 60 to 300 Hz) were deemed to be sufficiently accurate to determine the ICA. According to Duchowski et al. (2018), the details of the ICA implementation remain proprietary. Therefore, they developed an alternative open-source measure, the index of pupillary activity (IPA). Fehringer (2021) compared the IPA and ICA in 55 participants across four different tasks. The distributions of the ICA and IPA were similar. Blink rate can be determined easily with most eye trackers based on, e.g., the pupil size signal. For information on how to estimate blink rate, we refer to the section on blinks above. To our knowledge, there is only one video-based eye tracker that does report eye-tracking data even if the eyes are closed, namely the Pupil Invisible. However, Pupil Cloud^5^ (v5.7 and later) includes a signal that can be used in identifying blinks. Nyström et al. (2016) have demonstrated that saccade peak velocity has a complex relationship with pupil size in pupil-based eye trackers. For an intermediate pupil size, the peak velocity is greater than for both dilated and constricted pupils. This makes the eye-tracking metric “saccade velocity” not an attractive indicator for workload.

### Mind-wandering

Mind-wandering, or “zoning out”, occurs when someone involuntarily shifts their attention from the task at hand to task-unrelated thoughts (Smallwood & Schooler, 2006). Studies indicate that mind-wandering may constitute up to 50% of our waking hours (Killingsworth & Gilbert, 2010; Risko et al., 2012). To detect episodes of mind-wandering, researchers have primarily relied on self-reports using probe-caught and self-caught methods (Smallwood & Schooler, 2015; Varao-Sousa & Kingstone, 2019).

In the context of mind-wandering, eye tracking can be employed for different purposes. First, researchers may study how people’s eye movements differ during off-task compared with on-task behavior (e.g., Faber et al., 2020; Zhang et al., 2020). Second, eye-movement and pupil-size measures may serve as objective markers of mind-wandering during visual-cognitive tasks (e.g., Faber et al., 2018; Schad et al., 2012; Schad et al., 2024).

Since most eye-tracking research on mind-wandering has been conducted within the domain of text reading (see Steindorf and Rummel, 2020, for a review), this section will address mindless reading. To fully understand the following text, readers may wish to first consult the subsections “quasi-experimental approach”, “experimental approach”, and “data analysis” in the section “Eye tracking and reading” below. Cognitive control theories of reading assume a relatively tight link between the eye and the mind during normal reading (Reichle & Reingold, 2013; see also Hessels et al., 2025). During mindless reading, however, readers’ eye movements should be more or less decoupled from processing the text. To test this hypothesis, the quasi-experimental approach is particularly suitable.

Reichle et al. (2010) had four participants read an entire novel on a computer screen. Readers pressed a button when they caught themselves “zoning out” (self-caught method) and were occasionally prompted by the computer to indicate if they had been zoning out “just then” (probe-caught method). First-fixation durations and gaze durations were longer during mind-wandering (i.e., prior to self-caught and probe-caught zone outs) than during normal reading (i.e., prior to responding “no” to the probes). During normal reading, consistent with prior research, word frequency influenced various word-based measures of fixation time. During mindless reading, however, there was no word-frequency effect on first-fixation durations and gaze durations, indicating that lexical processing did not affect the decision of when to move the eyes. Building upon this work, Foulsham et al. (2013) adopted the experimental approach by presenting single sentences containing high- or low- frequency target words. Using the probe-caught method, the authors replicated the absence of the word frequency effect on gaze duration during mindless reading.

Schad et al. (2012) replaced subjective self-reports with an objective measure of mind-wandering. They operationalized mindless reading as failure to detect errors at different levels of text processing. Participants read stories from elementary school textbooks. During episodes of mindless reading (i.e., prior to overlooking errors that were inserted in the text), fixation durations were not elevated, different from Reichle et al. (2010) and Foulsham et al. (2013). For long words, the word frequency effect was significantly reduced during mindless reading compared with normal reading. More generally, the results by Schad et al. (2012) suggest that, during mind-wandering, cognitive processing becomes decoupled from external input in a graded manner, rather than all-or-nothing.

## Eye tracking to investigate eye movements that serve as a proxy

The aim of the current section is to quickly acquaint the novice eye-tracking researcher with useful operationalizations. Yet, what may be a useful or intuitive operationalization to one researcher may be very new to another. We have taken inspiration from our experience as instructors of various eye-tracking courses. Many participants in these courses are early-career researchers with diverse academic backgrounds (e.g., art history, experimental psychology, philosophy, cognitive science, physics, biology, medicine, engineering). Some participants have previously taken courses in experimental design or empirical research methods (for instance, psychologists or biologists). However, we also encounter participants who have never followed a course of this nature (e.g., individuals with educational backgrounds that lack a strong empirical component, such as philosophy, art history, or various design disciplines). The latter category of early-career researchers may hold naïve ideas about conducting empirical research with an eye tracker. An example of such an idea is that mere observation with an eye tracker is a sufficient way to investigate cognition. While we are not opposed to observation as a research method, certain research questions involving an eye tracker may require more than observation alone.

To further assist the broad readership of this article in designing research using eye tracking, we highlight two branches of research where eye movements serve as a proxy for key aspects of human behavior and cognition. The first pertains to reading research, a long-established field in eye tracking with well-established empirical methods, advanced analytical techniques, and consistent terminology. The second branch encompasses various applied research areas using an eye tracker. This research can pertain to human decision-making in the broadest sense, involving conscious decisions, such as: Does this mammogram contain a tumor? Or, which food product is more appealing? It may also concern unconscious decisions at the level of the visual or attentional system. An example of such a question is: How do advertisements distract from search behavior on a website or in a magazine?

### Eye tracking and reading

Let’s imagine, as a researcher, you are approached by Robin, a senior manager from the marketing department of a publishing house. She is interested in how their (online) journals and newspapers are consumed and can be better adjusted to specific customer groups. She has a lot of questions, e.g.: Do people with a university degree read a newspaper differently than people without? Does reading a magazine on an iPad differ from reading a physical magazine? Do older people read in a similar way as adolescents? These seemingly simple questions may be difficult to answer.

Approaching reading from a behavioral perspective, a fruitful approach involves analyzing gaze behavior during reading. The operationalization of reading may involve many observable aspects of gaze behavior (see section on data analysis below). Ultimately, eye tracking serves as a valuable tool for investigating the questions posed by Robin, drawing upon decades of basic research conducted in laboratory settings to advance our understanding of reading behaviors (Liversedge et al., 2022; Radach & Kennedy, 2004; Rayner, 1998).

**Fig. 3:**
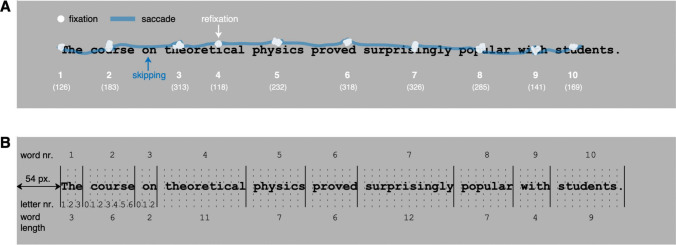
An example sentence. In panel **A**, a reader’s raw gaze data are superimposed, and panel **B** shows the corresponding physical layout of the sentence. In panel A, the numbers indicate the order of fixations whose durations are given in parentheses. In panel B, letter numbers are only provided for the first three words. The punctuation mark contributes to the length of the final word in the sentence

**Fig. 4:**
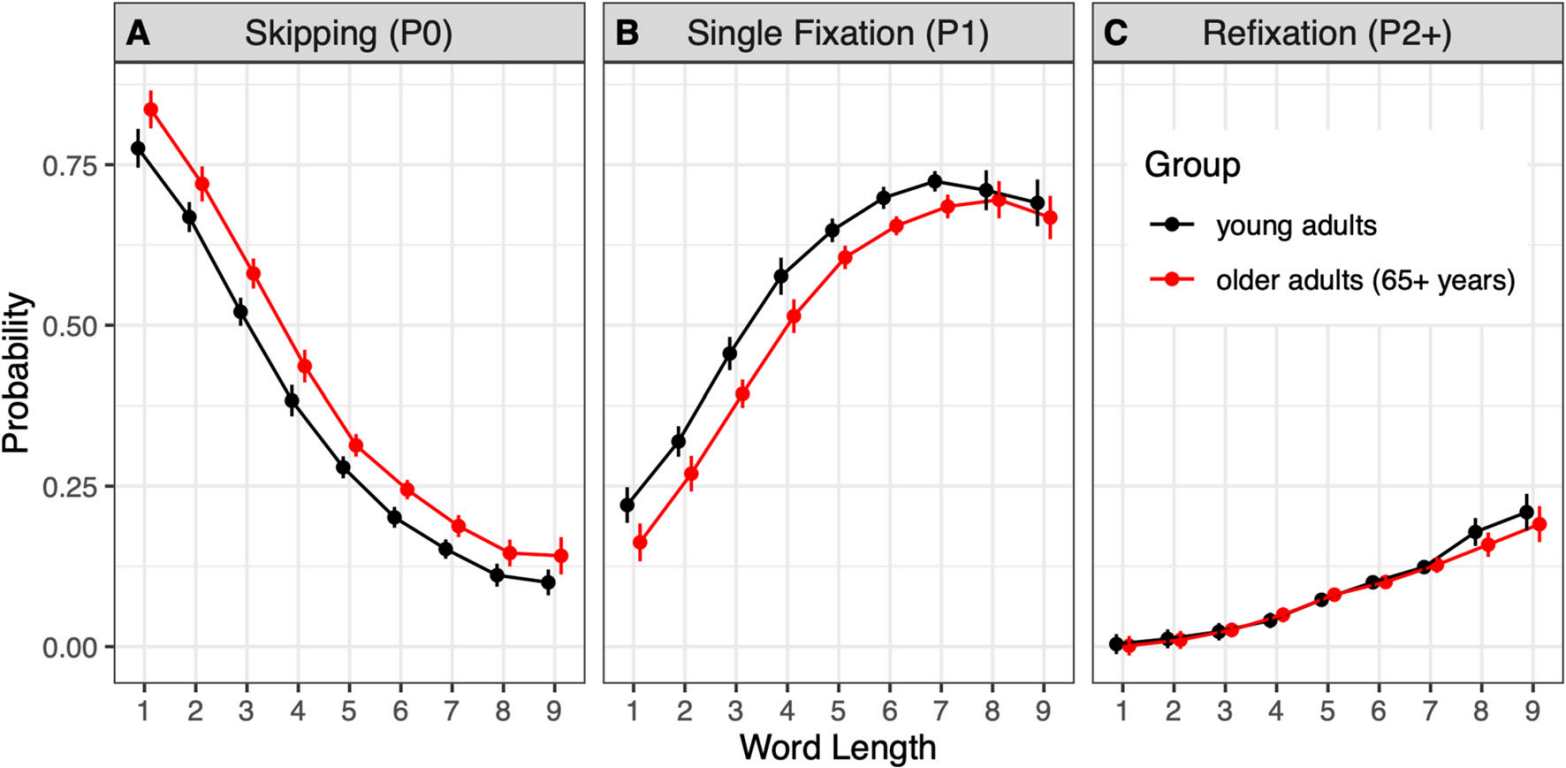
Word-based fixation probability measures for first-pass reading as a function of word length (in number of characters) for young (*black*) and older adults (*red*). Note. *Error bars* represent 95% CIs for mixed designs (Cousineau et al., 2021)

#### Quasi-experimental approach

Robin is intrigued to learn that readers do not look at each word in a strictly serial fashion, with one fixation per word (Fig. 3A). She wants to know how often readers deviate from such a pattern and whether older adults differ from young adults in this regard. Her intuition is that short words, like function words, may be skipped more often, while older readers may exhibit lower rates of skipping. Just as age is a property of people, length is a property of words, making both variables observational rather than experimental (Kliegl, 2007). To answer Robin’s questions, we may therefore conduct a quasi-experimental study where we record eye movements while participants read a corpus of natural text, consisting of individual sentences or passages (e.g., Kliegl et al., 2004; Siegelman et al., 2022). For demonstration purposes, we use data from an eye-tracking study involving 42 young adults and 34 older adults who completed two tasks, a scene viewing task (Nuthmann et al., 2020), and a reading task (unpublished). In the latter task, participants read 150 single sentences, including the one shown in Fig. 3. In the word-based analyses presented in Fig. 4, all words in each sentence, except for the first and last, were included. The data indicate that short words are frequently skipped and rarely refixated. As words become longer, skipping probability decreases and refixation probability increases. Contrary to Robin’s intuition, older adults exhibit higher skipping probabilities than young adults (Fig. 4A), aligning with previous research using alphabetic scripts (Zhang et al., 2022, for a meta-analysis). Although we do not report inferential statistics here, we note that the current standard is to analyze such data with (generalized) linear mixed-effects models (Baayen et al., 2008).

#### Experimental approach

While corpus analyses offer valuable insights, another approach commonly used to investigate eye movements during reading is to develop sentences or brief text passages containing specific target words or regions (Rayner et al., 2007). The objective is to manipulate variables related to these target elements and to observe how they influence readers’ eye movements.

Let’s assume one wants to find out how reading can be facilitated by using simpler words. We can investigate this question experimentally by constructing sentence frames containing a target word whose frequency–how often it occurs in English–we manipulate. Given the negative correlation between word frequency and length, where infrequent words tend to be longer, we need to control for word length (Kliegl et al., 1982). This is precisely what Rayner and Duffy (1986) did, whose participants read sentences with either low-frequency or high-frequency target words that were matched for word length. For instance, ‘The exhausted **steward** left the plane.’ represented the low-frequency condition, while ‘The exhausted **student** left the plane.’ represented the high-frequency condition. Both first fixation duration and gaze duration were significantly longer for infrequent than for frequent target words. In a way, these results indicate that using simpler (i.e., higher-frequency) words may indeed facilitate reading.

When using the experimental approach, reading researchers often employ gaze-contingent stimulus manipulations such as invisible boundary, moving window, parafoveal magnification, moving mask, text-onset delay, and disappearing text paradigms. These techniques allow for investigating specific aspects of cognitive processing during reading, such as the extent to which lexical and semantic information is processed outside foveal vision (Rayner, 2014; Rayner and Reingold, 2015; Schotter et al., 2012; Vasilev and Angele, 2017, for reviews).

#### Data analysis

Eye movements during reading can be analyzed at different levels, including paragraphs, sentences, phrases, words, and even individual letters within words. At the sentence level, typical measures include the total number of fixations, the average fixation duration, and the average length of right-directed saccades (e.g., Liversedge et al., 1980).

A common unit of analysis is the word (Inhoff & Radach, 1998). During the first encounter (or ‘pass’), a word can either be skipped (i.e., not fixated), fixated exactly once, or fixated more than once. Through data averaging, this gives rise to the probabilities of word skipping (P0), single fixation (P1), and refixation (P2+), with their sum totaling 1 (or 100%); see Fig. 4. While first-pass reading involves forward saccades, inter-word regressions move the eyes back to previously encountered text. Regression behavior can be captured by calculating the probability of a word serving either as the origin or the goal of a regressive saccade (Kliegl et al., 2004).

The spatial word-based probability measures are complemented by a wealth of temporal measures (Inhoff & Radach, 1998; Rayner, 1998; Starr & Rayner, 2001), including single fixation duration (SFD), first fixation duration (FFD), gaze duration (GD), and total fixation time. Typically, SFD, FFD, and GD are calculated conditional on the word being fixated on the first pass through the text (Rayner, 2009a). First fixation duration denotes the duration of the initial fixation on a word, regardless of whether it was refixated or not (Rayner, 1998). Gaze duration is obtained by summing the duration of the initial fixation on a word (FFD) and the duration(s) of any subsequent refixation(s) on that word prior to the eyes leaving it. Single fixation duration can only be calculated for words that receive exactly one fixation. Finally, total fixation time is derived by cumulating all fixation durations on a word, regardless of the reading pass. Naturally, the word-based eye-movement measures are interrelated to some degree. For example, increases in refixation probability are mirrored by longer gaze duration. Conversely, when words are not refixated, SFD, FFD, and GD are all identical. Therefore, when analyzing multiple eye-movement measures, researchers should consider corrections for multiple comparisons (von der Malsburg & Angele, 2017) and avoid selectively reporting results for only those measures that show significant effects.

#### Additional methodological considerations

To simulate natural reading conditions, one might consider presenting black text on a white background using a proportional font and standard line spacing to make efficient use of available space. However, this approach may not be optimal for investigating eye movements during reading.

In eye-tracking experiments, the text is often presented in a fixed-width font, also known as monospaced or non-proportional font (e.g., Courier New), where each character occupies the same width. The advantage to using a fixed-width font is that the word’s length, measured by the number of characters, directly corresponds to its visual width in degrees of visual angle (Rayner et al., 2010). This relationship is clarified by calculating the number of characters that subtend 1$^\circ$ of visual angle, a value that depends not only on the physical character width (in pixels) but also on the display size and viewing distance in the experiment (Carter & Luke, 2020; Hutton, 2019). Regarding the size of the font, it should be large enough to accommodate two factors: readability (Legge & Bigelow, 2011), and enabling reliable fixation-to-word assignment with the eye tracker used (Nyström et al., 2025).

Many studies on eye movements in reading have employed single sentences, each presented on a single line vertically centered on the monitor (e.g., Schotter et al., 2014). With this setup, assigning eye fixations to words and letters within words becomes straightforward (see Fig. 3B). The process can be performed algorithmically with scripting languages such as R, Python, or MATLAB, requiring just the horizontal position of the sentence on the monitor (54 pixels away from the left edge) and the character width (nine pixels), along with the actual text. In Fig. 3B, the solid vertical lines denote the word boundaries, while the dotted lines represent the letter boundaries. Considering that readers do not always fixate precisely on the line of text (Nuthmann, 2013), it is advisable to use AOIs that do not have a fixed vertical extension. When assigning fixations to text, the space between two words is usually regarded as letter 0 of the word that follows it, without adding to the word’s length (McConkie et al., 1988).

Complexities arise when stimuli consist of passages spanning multiple lines of text (e.g., Kliegl et al., 1982; Schad et al., 2012). In this case, readers do not only execute return sweeps from the end of one line to the beginning of the next (e.g., Parker and Slattery, 2019), but may also skip and/or revisit lines. Additionally, vertical drift may occur, potentially requiring post hoc correction of eye fixation locations (Carr et al., 2022). To improve the accuracy of assigning reading fixations to lines within a passage (Špakov et al., 2019; Tang et al., 2012), it is advisable to conduct the experiment with line spacing that is greater than the typical 1.2 to 1.5 times the font size used in printed text. This is particularly important when using an eye tracker with limited spatial accuracy (Shamy & Feitelson, 2023).

#### Computational modeling

In the late 1990s, sophisticated computational models of eye-movement control in reading began to emerge, embodying formalized versions of scientific theories (Reichle, 2021). The models are typically evaluated on their performance with benchmark data and their ability to make unique predictions (Rayner, 2009b). A lesser-known feature of computational models is their ability to help researchers explore phenomena that cannot be directly observed through eye tracking. For example, research on eye movements during reading is often based on the implicit assumption that saccades always land on their intended target words. However, distributional analyses of within-word fixation positions indicate that the eyes sometimes miss the intended word (McConkie et al., 1988). While the prevalence of these mislocated fixations can be estimated from empirical data (e.g., Nuthmann et al., 2005), it is not possible to classify individual fixations accordingly. Simulations with a computational model, however, provide data on the intended target word and actual landing position of each saccade. By comparing these outputs, researchers can identify instances where a saccade fails to land on its intended target (e.g., failed word skippings) and investigate how the oculomotor system responds (Engbert et al., 2007).

### Eye tracking for studying explicit and implicit decision-making

This section is about eye tracking and the question of how, as a researcher, one can sensibly study a wide range of topics with an eye tracker. At the operational level, much eye-tracking research in cognitive science, psychology, marketing, usability, health and education (not an exhaustive list) involves cases where the participant implicitly (e.g., preferential looking) or explicitly (button press) makes a decision. According to the eye-mind hypothesis (Just & Carpenter, 1976), an influential theory that connects eye movements to mental processing, 1) the unit of analysis is the gaze, which may consist of one or more fixations, 2) the locus of fixation may indicate what part of the visual stimulus is processed, and 3) the duration of the gaze is an indication of the encoding and processing phase. Before assuming such direct connection between detailed gaze behavior and mental processing, we advise to read Viviani (1990), Anderson et al. (2004) or Hessels et al. (2025). In practice, the interpretation of fixation locations and durations may be more difficult than expected. Obtaining longer fixation durations in experimental condition A than in condition B does not necessarily mean that mental processing takes more effort in condition A. It may also indicate that the observer likes the depicted flowers in condition A better and keeps fixating them. We also cannot exclude that the observer looked longer at certain locations for no specific reason et all. The assumption that fixation location and locus of covert attention coincide may be true in many situations, but cannot be assumed either (Posner, 1980; Deubel & Schneider, 1996). Being able to interpret fixation locations and durations can be facilitated by choosing the right combination of stimuli, instructions and keeping track of the participant’s other behaviors and performance.

#### The input–output model

In order to address the issues and solutions regarding how meaningful research with an eye tracker can be conducted, we resort to an old metaphor from cognitive science, namely the information processor (Marr, 2010; Dawson, 1998; Miller, 2003). We do not adopt this approach because we endorse this theory or hold a specific opinion on cognitive science as information processing. Rather, we employ this metaphor because we believe that the concept of an information processor serves as a useful framework for discussing empirical research with an eye tracker. By systematically describing the input, output, and state of the information processor, we can better predict the success of an experimental approach. The IO-model also helps in making decisions to enhance the effectiveness of experiments. Our focus is not on the human, mind, or brain as an information processor; instead, we emphasize the information content of the input and output and how it may relate to the internal state of the information processor.

The primary objective of our information processor metaphor, the IO-model, is to ascertain, during the design phase, whether an experiment encompasses sufficient information (e.g., in the stimuli and the instruction) to draw conclusions regarding, e.g., eye movement behavior, or the internal state of a participant (or group).

The IO-model comprises five aspects:

1. Task instruction (input): Examples of instructions are: Search for a target; Decide if a visual display contains a vertical green line; Choose one of two displayed shampoos; Follow the moving square with the cursor; Determine which of the two displayed patches is darker; Determine whether the chest x-ray contains a tumor. The instruction should be unambiguous.
2. Stimulus (input): Examples of stimuli are: A physical object in the lab; An image on a screen; A film clip projected on the wall; All previous visual stimuli but now with sound; A sound located on the right side of the participant. It may be important to validate the information content before using the stimuli. In the case of eye-tracking research using a wearable eye tracker, the stimulus may consist of the entire visual field of the participant. For an elaborate discussion about the concept of stimulus see Gibson (1960).
3. Behavior (output): Examples that can be measured with an eye tracker are: amplitude of the saccade, fixation duration, number of fixations, number of microsaccades, number of refixations of a specific location, the number of words skipped, curvature of a saccade etc.
4. Performance (output): Examples of performance include the number of correct responses, $d'$, reaction time, speed of the movement, the proportion false alarms, occurrence and number of specific errors. There exist various methods for interpreting performance: 1) by comparing it with the performance of a baseline measurement, 2) by comparing it with the outcome of a theoretical model, 3) by referencing a benchmark database (typically used in marketing research), or 4) by comparing it with performance observed in previous studies documented in the literature.
5. (Optional) State or Internal state: Examples of internal state are descriptions of the participant or participant group. One may think of personality traits (e.g., big five score), emotional state, expertise level (e.g., novice, expert), gender, prior knowledge, description of a condition such as ADHD or OCD.

Why is it important to record performance? For interpreting the variation in behavior among distinct participants or participant groups, it may be advantageous to eliminate the possibility of different strategies being employed. If different strategies have indeed been employed, it is useful to identify the specific strategies in use. For instance, a performance metric may reveal the existence of a speed-accuracy trade-off (Salthouse, 1979; Heitz, 2014). Furthermore, performance metrics serve as a valuable tool for assessing participants’ comprehension of instructions and their ability to execute tasks. In essence, performance measurement may help improve the interpretation of (eye movement) behavior.

Of course, the IO-model is a very simple approximation. To explore whether this approach is fruitful, this section discusses three prototypical experiments that can serve as prototypes for many more experiments to be conducted. What these three prototypes have in common is that they all involve visual stimuli, some form of decision-making task (in the broadest sense) and gaze behavior. We will use the IO-model in the following way. Let’s consider the following proposal for an eye-tracking experiment from the field of history. The research question is: How did medieval people look at graffiti? To investigate this question, the researchers will use VR-goggles with a built-in eye tracker. In their research proposal, we can identify a visual stimulus (a detailed virtual environment of the Utrecht St. Martin’s Cathedral), an aspect of eye movement behavior (AOI statistics of medieval graffiti on the walls of the church), and a description of the participant group (25 second-year university students). Without delving into details about this study, it is apparent what is lacking, namely an instruction and a performance measure. We would advise the researchers to think about an instruction for the participants. If the researchers cannot come up with a performance measure, we ask for a sensible baseline for comparison of their obtained behavior. The previous example illustrates that a free-viewing study is often not an effective method for drawing conclusions based on the observed eye movement behavior. In many free-viewing studies, it is not clear what the participants do and how well they do it.

**Fig. 5:**
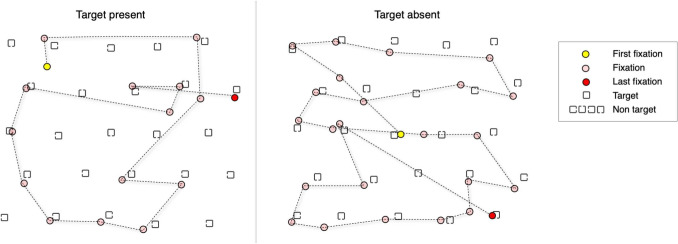
Search displays. A target-present trial and a target-absent trial. The target is a closed symbol (O), the non-target is a C appearing in four orientations. The first fixation is indicated by a *yellow circle*. The last fixation is indicated by a *red circle*. Note, in this example, the target and non-targets are black on a white background. In the original experiment (Toffolo et al., 2013), they were white on a gray background

#### Decision-making in a controlled experiment: Search task and eye tracking

Godwin et al. (2021, p. 2753) wrote: “visual search is used as a tool by researchers to study a broad spectrum of different “basic” aspects of cognition and information processing”. We often advise master students and novice PhD candidates: “If you can operationalize your research question as a search task, do so, as it will make your life much easier”. This may sound as a random and unmotivated advice, but it should be seen as a heuristic rule when no specific other plan is there. Why? When eye tracking and a search task (or a signal detection task) are used together, the requirements of the IO-model are met. For instance, a master student may wish to investigate which peanut butter packaging stands out most prominently within the context of a supermarket shelf. *Standing out* in the shelve can be operationalized as *easiest to find* among other products and quantified using time to first fixation, or a manual reaction time. This research includes clear instructions (search for the peanut butter), a visual stimulus (an image of the peanut butter among other products), observable behavior (e.g., number of fixations, saccade amplitude, and number of refixations on a product), and performance metrics (search time, number of successful trials where the peanut butter was quickly found). The metrics are interpretable because there is a vast literature of (visual and memory) search, and the characteristics are well known (Van Zandt & Townsend, 1993; Sternberg, 1969; Gilchrist & Harvey, 2000; Eckstein, 2011; Wolfe, 1998). Furthermore, in many cases, signal detection theory (SDT) can be applied to produce additional performance (e.g., sensitivity) and behavioral measures (the decision criterion). For readers unfamiliar with SDT, we recommend the article by Stanislaw and Todorov (1999), which provides a good summary of SDT, including examples in Excel and MATLAB to calculate a variety of signal detection measures.^6^ Additionally, we recommend Wright et al. (2009).

The following paragraph is about applying knowledge on the serial search task (Van Zandt & Townsend, 1993; Sternberg, 1969) to predict, compare and interpret performance in an eye movement search task. The classic search task has a target-absent and a target-present condition (Wolfe, 1998). For this example, let’s assume a search display with 25 elements loosely placed on a hexagonal grid (see Fig. 5). In such a grid, the distance between all objects is similar in all directions. In the present case, the target is a closed object (a square-like O), and the non-target is a C that can appear in four orientations. If the search display is constructed in a way that only the fixated object can be properly evaluated, then in a target-absent display, 25 fixations are required to decisively determine that no target is present in the display because all objects must be inspected. In a target-present display (from a condition where targets are homogeneously distributed across all locations in separate trials), the target may be found in the two extreme cases either on the first fixation or the last fixation if the searcher does not revisit locations. In this case, we expect the target to be found on average after 12.5 fixations. In an eye movement search task the number of fixations usually has a high correlation with the reaction time (search time). Therefore this example can be seen as ‘the number of fixations’ analogon of the ‘standard serial processing hypothesis’^7^ (Van Zandt & Townsend, 1993; Sternberg, 1969). If the participant can evaluate more objects per fixation (which is hard to exclude in experimental settings), we expect fewer fixations than predicted above, but the number of fixations in the target-present condition should still be half that of the number of fixations in the target-absent condition. Deviations from the theoretically predicted number of fixations are interesting and may indicate different mental processing, different perception and lack of memory for already inspected objects. Here the number of fixations may serve as a proxy for other concepts.

Operationalizing a research question as a search task can work well when the topic is “search-like”. Searching for information or objects is one of the most common human behaviors. Topics from user experience (UX) and the medical field often lend themselves well to the visual search approach. The search approach can also be effective when there is an expectation that individuals with a certain condition (e.g., ADHD, PTSD , OCD) or in a particular state (e.g., emotionally) behave differently than controls. This makes the application of the visual search approach valuable in clinical psychology.

An example of applying this general knowledge about the number of fixations in a search task is the study by Toffolo et al. (2013). The subject of the study was the uncertainty of individuals with OCD. How to investigate whether people with OCD make different decisions in an uncertain situation? The study compared the search behavior of people with high obsessive-compulsive tendencies (OC+) to those with low obsessive-compulsive tendencies (OC-). People with OCD are known for excessive checking behavior when uncertainty is involved. Participants viewed 50 visual search displays, with 25 target-absent and 25 target-present trials (Fig. 5) presented in random order. They indicated whether a target was present or absent. Decisions about the presence of the target caused little uncertainty because participants could visually verify the target. In the target-present condition, search times and the number of fixations did not differ between the OC+ and OC- groups. In target-absent trials, OC+ participants took longer to search and produced more fixations than OC- participants. Thus, the number of fixations and search times produced qualitatively similar results. However, concluding that eye tracking provides no additional benefit in this context would be premature. More fine-grained analyses could have explored whether the two participant groups differ in scanning time and verification time, as inferred from eye movements during target-present trials (e.g., Nuthmann and Malcolm, 2016) and in their fixation selection strategies.

Another example of operationalizing the research question as a search task is the study of pull-down menus in computer interfaces by Byrne et al. (1999). The study aimed to provide data that could help refine the understanding of how people interact with pull-down menus. Participants first saw a screen with a rectangle containing the word “Goal” followed by a target symbol. When the user clicked on this rectangle, a pull-down menu with symbols appeared. Users then searched for the target item in the menu and clicked on it. Gaze and cursor positions were recorded, and response time and accuracy were documented. Participants typically searched from top to bottom, inspecting items sequentially (not randomly), and sometimes skipped items.

The last example of operationalizing the research question as a search task is the study by Kok et al. (2016) on systematic viewing in radiology. Radiology textbooks recommend “systematic viewing”, a technique where anatomical areas are inspected in a fixed order. This systematic viewing is expected to enhance diagnostic performance. To test this assumption, two experiments were conducted to investigate the relationship between systematic viewing, coverage, and diagnostic performance. Participants included novices and experts, with a portion of the novices receiving “systematic search training”. By employing a search task Kok et al. (2016) revealed that experts inspected cases more systematically than novices, and that novice students did not benefit from systematic viewing training.

**Fig. 6:**
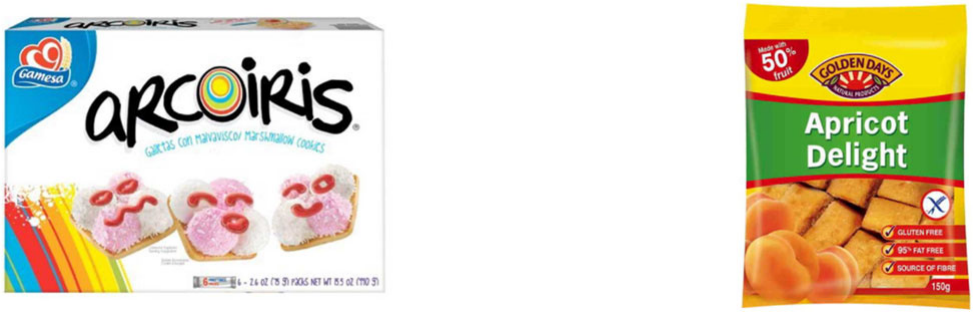
Food product choice. The participants were asked to choose one of the two products. For presentation purposes, in this article we choose a white background instead of a gray background in the study of van der Laan et al. (2015)

#### Decision-making in a less controlled experiment: Choice test and matching

Imagine a food product choice test with eye tracking. Does a choice test meet the requirement for the IO-model? Let’s begin with the inputs. The instruction to the participant is clear: “Choose the product that you want to have by pressing the left or right arrow key”. An example of a stimulus display for a food product choice test is shown in Fig. 6. The visual stimulus can be well described in terms of, e.g., size, color, familiarity, and product category. However, it is not clear beforehand how these properties may affect the decision process. In contrast, in visual search, the relation between many properties of the visual stimulus and the RT is well described in the literature (e.g., target-distractor dissimilarity is an important factor, Duncan and Humphreys, 1989; Pashler, 1987; Treisman and Gelade, 1980). Regarding the output, behavior (gaze and a keypress) can be recorded and an AOI analysis can be applied to the gaze data. However, there is no performance measure. This may be problematic for understanding how the task was conducted, because the participants may use different strategies to choose a product (e.g., the biggest, the one with milk or the one without fat). Compared to the search task, the researcher has less knowledge of the stimulus material, because of the lack of a theory on the preference of food products. Also, the lack of a performance measure makes it more difficult to check whether the participants used different strategies or not.

Before delving into the more complex topics, let’s first describe the type of research questions regarding the choice experiment that are straightforward to operationalize. If the research question is about the influence of a visual aspect (e.g., the size or roundness of the product) on the choice, then the size or roundness can be varied, and the impact on how often the product is chosen can be examined. When an experiment involves only two or three conditions, this solution is feasible. When the number of conditions is small, one can also apply a factorial design to study interactions.

However, when research questions revolve around the role of eye movements or fixations in decision-making, it becomes a different story. Do people choose what they look at, or do they look at what they choose? If one wants to know the influence of the first fixated object on the decision (as in van der Laan et al., 2015), one is not interested in the visual properties of the food stimuli and does not want them to play a complicating factor in the choice test. In this instance of applied research, the packaging also needs to be representative of real packaging, resulting in significant variation in color, size, and shape among the packagings. Due to the high degree of variability in packaging, the implementation of specific experimental conditions may therefore not be feasible.

Instead of having many conditions, one may try to neutralize most of the factors that could influence the choice. Is that possible? In a choice task, Schotter et al. (2010) investigated how much of the gaze bias effect is due to a liking effect (Shimojo et al., 2003), compared to the information encoding aspect (Glaholt et al., 2009) of the decision-making process. Schotter et al. (2010) asked participants to choose between two photos. They ensured that within pairs, the photos were matched for content (e.g., both portraits, both landscapes) and for color content (both color or both black- and-white). Van der Laan et al. (2015) went a step further with matching of attractiveness of both alternatives in the food product test. They applied a method aiming to render all factors related to attractiveness ineffective (including the unknown and participant-dependent ones). Before participants conducted the actual choice test, they were asked to rate all products. Based on the ratings, a personalized stimulus set was created for each participant, with each stimulus display (as in Fig. 6) consisting of two products that were equally attractive (had the same rating) for the respective participant. This way, an attempt was made to eliminate the influence of most factors in the choice. We recommend that anyone interested in the decision-making process who is not directly interested in the influence of specific aspects of the stimulus material should use a matching procedure to produce (individualized) stimulus sets.

#### Need for control: Constructing a baseline condition

From the search task example, it becomes clear that if the researcher has knowledge about the relation between stimulus material, instruction, behavior and the performance, a potentially effective experiment to study the decision-making process can be conducted. From the choice example, it becomes clear that despite the lack of a performance measure, stimulus matching is a good method to conduct effective research on the role of eye movements in the decision-making process. In the following example, we take it a step further again. Suppose the context is marketing research or media studies and the research question for the study is: What is the attention-attracting power of advertisements on web pages? In marketing research, the answer to such a question can be used to optimize advertisement location, while in media studies, it can be used to investigate how children are distracted by ads on child-oriented websites (as in Holmberg et al., 2014).

Let’s start with applying the IO-model. What would the instruction to the participant be? This is already a difficult question; some researchers are hesitant to provide explicit instructions because they believe that such instructions may influence the natural behavior of participants, which they wish to avoid. We posit that an instruction such as “explore this website as you would do at home while sitting on the couch with your tablet” may be more appropriate than giving no instruction at all. Keep in mind that not giving an explicit instruction means that the instruction is undefined, not that there is no instruction or expectation of the participant. In the case of a webpage, the content may include both static and dynamic materials, and if participants are free to browse and click, the stimulus material varies among individuals. The researcher must make clear choices here. This decision likely revolves around the representativeness (Holleman et al., 2020) of the test website versus the practical execution of the test. The output consists of gaze data, which again can be analyzed using AOIs, while a performance measure is absent. Because there is no performance measure, in the light of the IO-model, it seems unwise to utilize the eye tracker as an observational tool during a complex task as browsing webpages aimed at investigating how advertisements attract attention.

From previous research, it is known that the attention-attracting power of an advertisement depends on multiple factors. First, there are image factors, such as the advertisement’s location on the page, its size, the white space around it, and the color contrast with its surroundings (not an exhaustive list). Additionally, there are factors associated with the participant’s instruction (or intention or task). In the context of marketing research and media studies, participants are primarily engaged in activities like reading an online newspaper, seeking information, or playing an online game. During these activities, they (unconsciously) may not focus on locations where ads typically appear. This phenomenon is known as one of the explanations for *banner blindness* (Benway, 1998; Owens et al., 2011; Resnick & Albert, 2014; Lee & Ahn, 2012).

The last factor we want to discuss pertains to looking biases. When individuals make a saccade to an object, they tend to land on or near the center of mass (Melcher & Kowler, 1999; Vishwanath & Kowler, 2003). In photorealistic scenes, viewers prefer to fixate on objects, with the preferred viewing location (PVL) being close to the center of objects (Nuthmann & Henderson, 2010; Pajak & Nuthmann, 2013). When observing framed scenes such as computer monitors, paintings, and magazines, people tend to display a central bias by fixating predominantly in the center of the frame (Tatler, 2007; Tatler & Vincent, 2009; Stainer et al., 2013). Using the aforementioned factors, one can reason about which advertisements are more likely to capture attention and which are less likely to do so. However, determining the attention-attracting power of real advertisements on a web page is theoretically challenging. The answer to this question can be empirically approached through a factorial design, considering all combinations of advertisement size, color, amount of white space, color contrast with the background, and other stimulus factors, along with varying instructions given to participants. However, conducting such an experiment is impractical and demands considerable time and effort. Nevertheless, attempts have been made to assess the attention-attracting power of advertisements using similar methods, including studies on outdoor ads (Wilson & Casper, 2016; Crundall et al., 2006), newspaper ads (Simola et al., 2013), and banners (Lee & Ahn, 2012).

**Fig. 7:**
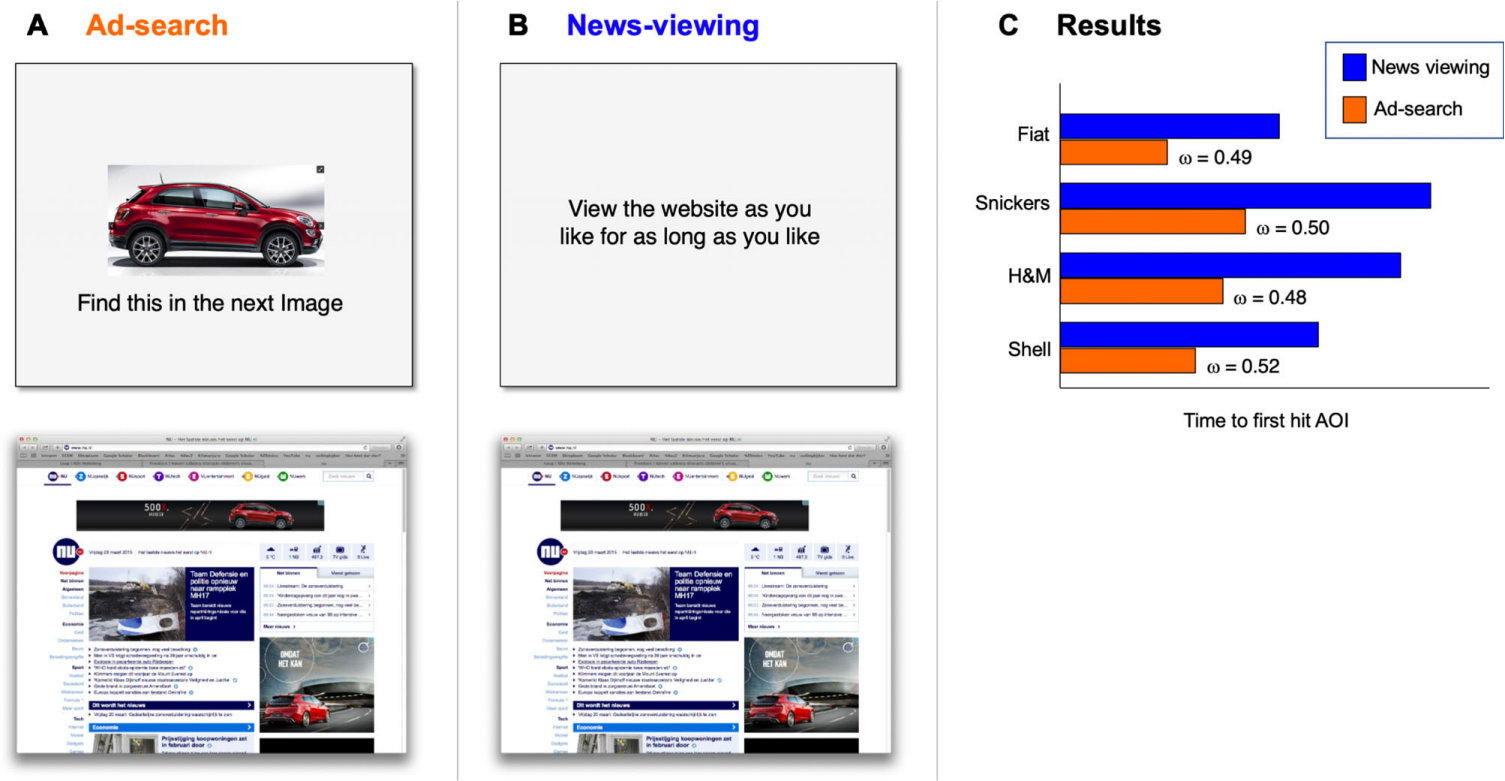
Ad search. Panel **A** contains the condition where the attention-grabbing power of a specific advertisement is estimated. The task given to the participant is to find the image of the red Fiat. Panel **B** contains the condition where participants, for example, look at the displayed pages as they wish (in this case, reading the news). Panel **C** depicts the time to first fixation of the AOI (TFF) of the advertisements for the search and news-viewing conditions. In panel C, it can be seen that in this case, the advertisements have much longer TFFs in the news-viewing condition compared to the advertisement search condition. A longer TFF points to less attention-attracting power of the advertisement. A measure of distractibility is the quotient ($\omega$) of TFF_search_ and TFF_news_. The values for $\omega$ are lower than 1 and indicate less distraction by the advertisements in the news-reading condition than the baseline (search) condition. Disclaimer: This figure depicts a thought experiment for which the data were produced by the authors

Assuming that we are not specifically interested in image factors (e.g., location, color, amount of space around the advertisement) but rather in the original question about the attention-attracting power of advertisements on a website, we propose a pragmatic approach to address this issue. Firstly, we need an operationalization of the attention-attracting power of an advertisement (see section on attention-attracting power above). For this purpose, we consider the time until participants first fixate within the target-ad AOI. This time is referred to as the time to first fixation of the AOI (TFF). A practical method for operationalizing the TFF of a group of participants is the T_50_ (for an explanation, see Hooge and Camps, 2013). The shorter the T_50_, the stronger the attention-attraction power of an AOI. To be able to interpret the TFF of an advertisement or another visual element, a baseline is required. Only then can we determine whether the observed value is short or long in comparison to the baseline.

At this point, we do not yet have a baseline. How do we establish one? We could determine the attention-attracting power of each advertisement in its own context (e.g., newspaper, gaming webpage). Here we introduce a thought experiment consisting of two parts. In the first part, we determine the attention-attracting power for each advertisement by measuring the TFF (see Fig. 7 panel A). We do this by having participants search for the specific advertisements. This provides us with the maximum attention-attracting power (the minimum TFF) for that advertisement in that specific location on the page. In a second experiment, another group of participants read the newspaper page, and the TFF is estimated for each advertisement (see Fig. 7 panel B). The final step is to calculate the distractibility quotient ($\omega$), a measure that incorporates the baseline. This index is the quotient of TFF_search_ and TFF_news_. What does a certain value of $\omega$ mean?

Let’s discuss some values of the distractibility quotient. $\omega < 1$ means that the attention-attracting power of the ads is reduced during newspaper reading. This outcome is to be expected. A value close to zero for $\omega$ is an indication that participants are capable of completely ignoring the ads while reading. On the other hand, $\omega = 1$ means that the advertisement attracts attention with the same power during reading as during the ad search task. This indicates the highest attracting power of ads possible and is probably what an ad designer wants to achieve.

When a web page contains numerous advertisements, the proposed empirical approach may still incur significant research time. Then, it is advisable to consider using fewer or even only one advertisement to assess the attention-grabbing impact of advertisements while engaging in another task as such as reading, scene viewing or search for information.

## Conclusion

In this article, we have discussed operationalizations and examples of experimental design. We started with the observation that even seemingly measurable entities such as saccades may be subject to operationalization. Such operationalizations (often algorithmic) closely align with the original concept of the saccade, thus prompting the introduction of the term direct operationalization. Additionally, many other concepts are not directly measurable (e.g., attention-attracting power, or mind-wandering). To operationalize these, a concept that appears seemingly measurable (and hence operationalizable) must be selected. We have termed this indirect operationalization.

Furthermore, we have discussed that many implementations of operationalizations are not fixed. In the case of algorithmic operationalization, such as fixation, the parameters within the detection algorithm may play a significant role. As a researcher, one should understand that an operationalization can be changed by varying the parameters of a saccade detector or varying the size of an AOI. One of the aims of operationalization is to facilitate the comparison of data across different studies when the same operationalization is employed. Therefore, we advocate for transparency in publications regarding the parameters and algorithms utilized.

In this article, we have provided the reader with references to studies that report operationalizations of commonly used concepts in eye-tracking research. Moreover, we have discussed important operationalizations in reading research. In the last part of the article we have introduced the IO-model. This is a tool that may help researchers to operationalize difficult concepts in an experiment or observation with an eye tracker. The last part of the article consists of three prototypical example experiments with useful methods to conduct eye-tracking research (e.g., the search task, matching of stimuli, and producing a baseline). We advise the reader to carefully consider these three experiments, even if they are about topics other than their own, because they may serve as inspiration for experiments about other topics.

## Data Availability

Not applicable.

## Footnotes

1 SR Research Data Viewer is software for processing and visualizing gaze data recorded with EyeLink eye trackers, which are widely used in reading and vision research. Many EyeLink users use the SR Research data viewer software to classify saccades in the eye tracker signal.

2 Software to design an experiment, record gaze, and analyze eye-tracking data for Tobii eye trackers.

3 There are heatmaps, however, where fixations are weighted by fixation durations (see e.g., Henderson, 2003).

4 Response to the question “How much do you have a sense of understanding this artwork?”

5 Pupil Cloud is a web-based eye-tracking platform for data logistics, visualization, and analysis for Pupil Labs eye trackers.

6 Formula 5 in Stanislaw and Todorov (1999) contains an error, the rightmost 2 must be inside the curly braces.

7 Van Zandt and Townsend (1993, p. 564) wrote: “The standard serial processing hypothesis assumes that average processing times for all individual items are equal and independent of search set size, positions of items, and item identity. It predicts that the *set-size functions* (plots of mean RT as functions of the size of the search set) will be linear and increasing. If search is self-terminating as well, the positive set-size function will increase with a slope equal to half that of the negative set-size function. This is because the target will be found, on the average, half-way through the search, and processing can then terminate.”

## Declarations

### Open Practices Statement

There are no data or code associated with the present article.
